# Computable structured aptamer for targeted treatment of ovarian cancer

**DOI:** 10.3389/fgene.2023.1170260

**Published:** 2023-05-03

**Authors:** Luoshan Ruan, Liting Han, Xin Li, Xin Chen, Gege Sun, Xinyu Wang, Yan Luo, Chuanqi Gu, Xiaolong Shi

**Affiliations:** ^1^Department 2 of Gynecology, Remin Hospital of Wuhan University, Wuhan, China; ^2^ Institute of Computing Science and Technology, Guangzhou University, Guangzhou, China

**Keywords:** nucleolin, AS1411 aptamer, DNA tile, doxorubicin, ovarian cancer

## Abstract

Nucleolin protein expression is higher on the ovarian cancer cell surface. AS1411, a DNA aptamer, can bind with nucleolin protein specifically. In this study, we developed HA and ST DNA tiles to assemble six AS1411 aptamers to deliver doxorubicin. In addition, to superior serum stability and drug loading, HA-6AS and ST-6AS outperformed TDN-AS in cellular uptake. HA-6AS and ST-6AS exhibited satisfactory targeted cytotoxicity and achieved resounding lysosomal escape. Moreover, when injected into nude mice subcutaneous xenograft models, HA-6AS reached the peak in tumor more quickly than ST-6AS, and better expressed the active targeting ability of AS1411. Our study suggests that designing appropriate DNA tiles to assemble different aptamers to deliver different chemotherapeutic drugs is a promising treatment for ovarian cancer.

## 1 Introduction

Epithelial ovarian cancer has the highest mortality rate among gynecological malignancies (Lheureux et al., 2019b; Colombo et al., 2019; Kuroki and Guntupalli, 2020). On one hand, due to the lack of early diagnosis methods and specific symptoms, most patients are at an advanced stage when they are diagnosed (Broekman et al., 2018; Lheureux et al., 2019a; Walker et al., 2019; Govindarajan et al., 2020; Wang et al., 2021); on the other hand, traditional platinum-taxane is still the main chemotherapeutic agent for ovarian cancer (Pujade-Lauraine et al., 2010; Pignata et al., 2011; Gourley and Bookman, 2019; García-Martínez et al., 2020). However, because of its indiscriminate toxicity, patients often cannot tolerate the adverse effects of chemotherapy.

Studies are increasingly exploring targeted therapies for ovarian cancer. Aptamers, which are screened from a random single-stranded DNA or RNA library (Ellington and Szostak, 1990; Tuerk and Gold, 1990), have recently become popular for guiding delivery materials, such as Au nanoparticles (Amouzadeh Tabrizi et al., 2018; Kim et al., 2021; Yang et al., 2021), quantum dots (Kim et al., 2019; Zheng et al., 2019; Adegoke et al., 2020; Jamei et al., 2021), and organic polymers (Hashemi et al., 2020; Chen et al., 2021; Dai et al., 2021), to their intended location. In addition, aptamers efficiently bind to targets with high affinity and are low immunogenic (Wu et al., 2015; Chen et al., 2017; Hoosen et al., 2018; Zhu and Chen, 2018; Yan et al., 2021; Zhao et al., 2022). AS1411 is a 26-nt DNA aptamer with a G-quadruplex structure that binds to nucleolin protein, whose expression in several cancer cells is much higher than that in normal cells, and is located on the surface of ovarian cancer cells, whereas it is normally located in the nucleus of other cancer cells (Bates et al., 2017; Li et al., 2017; Yazdian-Robati et al., 2020; Tong et al., 2022). Therefore, AS1411 can be used as a target signal in ovarian cancer cells.

Since metallic nanoparticles, graphene oxide, and carbon nanotubes are toxic, the encapsulation efficiency and stability of liposomes are unsatisfactory, and extracellular vesicles are heterogeneous and difficult to preserve (Cheng et al., 2021); therefore, biomaterials with biocompatibility and biodegradation have been developed, such as DNA origami or tiles. The DNA tile is a basic assembly unit that uses single strand DNA with sticky ends based on the complementary base pairing principle (Evans and Winfree, 2017). Over the last 2 decades, it has become increasingly complex and diverse and can be used to construct more complicated 2D patterns and 3D structures (Shi et al., 2014)). Based on the DNA tile motif, a computable program to assemble several aptamers could be an efficient targeting drug delivery unit. In addition, doxorubicin (DOX, also known as doxorubicin) is a common chemotherapeutic drug in cancer. In 2018, the National Comprehensive Cancer Network (NCCN) guidelines included pegylated liposomal doxorubicin in the treatment of patients with initial treatment of ovarian cancer and patients with platinum-sensitive and platinum-resistant recurrent ovarian cancer. At the same time, DOX can be loaded into the DNA nanomaterials simply by intercalation; therefore, we explored the feasibility of using a structured aptamer loaded with DOX to cure ovarian cancer.

In this study, we assembled six AS1411 aptamers to two hexangular DNA tiles for the first time and constructed two efficient but different structured aptamers; HA-6AS, which is 3D tubular and ST-6AS, which has a 2D six-star structure. We discovered that HA-6AS and ST-6AS could load more DOX than DNA tetrahedron and deliver DOX into ovarian cancer cells, allowing it to escape from lysosomes and reach the nucleus. HA-6AS-DOX and ST-6AS-DOX exhibited rapid cytotoxicity in ovarian cancer cells but low cytotoxicity in normal ovarian epithelial cells. Furthermore, we demonstrated that the internalization patterns of HA-6AS and ST-6AS might be different; HA-6AS was more actively targeted, whereas ST-6AS was more passively targeted. We speculated that tubular HA DNA tiles may be more appropriate for the assembly of AS1411 aptamers. In summary, DNA tiles can be a useful biocompatible candidate for targeted drug delivery and cancer treatment.

## 2 Materials and methods

### 2.1 Cell culture

The SKOV3 and IOSE80 cell lines were purchased from the China Center for Type Culture Collection (CCTCC, China). SKOV3 cells were maintained in RPMI-1640 medium (Hyclone, United States) containing 10% fetal bovine serum (FBS, Gibco, United States) and 1% penicillin-streptomycin (Biosharp, China), and IOSE80 cells were cultured in DMEM (Hyclone, United States) with the same content of FBS and penicillin-streptomycin. All cells were incubated at 37°C with 5% CO_2_ and 95% humidified atmosphere.

### 2.2 One-step preparation of HA-6AS, ST-6AS and TDN-AS

All single DNA strands and AS1411 aptamers (with or without fluorophores) were synthesized and purchased from Sangon Biotech (China). They were normalized to 10 μM with 1× TAE buffer containing 12.5 mM Mg^2+^ and stored at 4°C. Equal volumes of HAs1, HAs2, HAs3, HAs4, HAs5, and HAs6 and 6-fold AS1411 were mixed together and self-assembled during a PCR annel program, which was set to a slow rate with a 1°C drop every 8 min after for 15 min at 95°C. The synthesis of ST-6AS was the same as that of HA-6AS. The anchored strands are referred to as the “handle.” For TDN-AS, equal volumes of handle-S1, S2, S3, S4, and AS1411 were mixed and then self-assembled during the same PCR annealing program. All structured aptamers were validated using 4% acrylamide gel electrophoresis. The branch strand sequences from 5′to 3′of the two DNA tiles and the DNA tetrahedron are described in the supplementary materials. The sequence of AS1411 with the anchored strands and 4 T spacer was as follows: Handle-4T-AS1411: TCT TCT TTC TTA CTT TTT GGT GGT GGT GGT TGT GGT GGT GGT GG.

### 2.3 AFM characterization of HA, ST, HA-6AS and ST-6AS

AFM imaging was conducted in Scan Analyst-fluid mode (Multimode 8, Bruker) or AC water topography (Cipher ES, Oxford). A 5 μL sample was dropped onto newly prepared mica, left for adsorption for 10 min, and then observed under a microscope.

### 2.4 TEM characterization of HA, ST, HA-6AS and ST-6AS

Ten μL sample was spotted onto a continuous carbon support film (Zhongjingkeyi Film Technology Co., Ltd., Beijing). After deposition for 10 min, the excess sample was removed, and 2 μL 3% uranyl acetate was dropped for negative staining for 1 min. The samples were analyzed using TEM (Thermo Fisher Scientific Talos F200C).

### 2.5 Serum stability assay

HA, ST, TDN, HA-6AS, ST-6AS, and TDN-AS were incubated in equal volumes of 10% FBS medium for different durations at 37°C. The samples were collected and frozen at −20°C. A 4% PAGE was used to visualize degradation.

### 2.6 DOX loading

HA-6AS, ST-6AS, and TDN-AS were mixed with an equal volume of DOX, and the mixture was incubated for 3 h at room temperature with gentle shaking before it was centrifuged for 20 min at 14,000 *g*. A MicroSpectrophotometer (KAIAO K5500Plus, China) was used to measure the concentration of dissociative DOX in the supernatant at 495 nm absorption with a 195 extinction coefficient.

### 2.7 Flow cytometry

HA-6(FAM-AS), ST-6(FAM-AS), and TDN-(FAM-AS) were diluted to different concentrations (62.5, 125, 250, and 500 nM and 1 μM). After 3 h of incubation, SKOV3 or IOSE80 cells were trypsinized and centrifuged (1,500 rpm × 5 min), washed twice with PBS, and resuspended gently in 150 μL PBS. The fluorescence intensity of FAM was measured with a Beckman CytoFLEX by counting 15,000 events.

### 2.8 Co-localization of DOX and DNA origami

DOX (1.5 × 10^4^) was seeded into 12-well plates and cultured overnight. Cells were incubated with HA-6(FAM-AS)-DOX, ST-6(FAM-AS)-DOX, or TDN-(FAM-AS)-DOX, which were added to a final concentration of 500 nM AS1411 in PBS buffer for 3 h. After washing three times with PBS, the cells were photographed using an inverted fluorescence microscope (Olympus IX71, Japan).

### 2.9 Cell viability assay

SKOV3 and IOSE80 cells (1×10^4^) were seeded into 96-well plates and cultured overnight. The cells were treated with various concentrations of AS1411, HA, ST, HA-6AS, and ST-6AS for 24 h to examine the toxicity of free AS1411 and structured AS1411. After that, SKOV3 and IOSE80 cells (1.5×10^4^) were seeded into 96-well plates and cultured overnight. For the cytotoxicity assay, DOX, HA-6AS-DOX, ST-6AS-DOX, and free DOX were diluted with complete medium to achieve a series of equidifferent concentrations from 6 to 30 μM, which were calculated using DOX. After 3 h of treatment, the cells were incubated for 2 h with CCK8. The absorbance was measured using a microplate reader at 450 nm. When the cytotoxicity assay with DOX was extended to 24 h, DOX was diluted in an equal ratio of 2.4 nM–24 μM.

### 2.10 Confocal imaging for lysosomal escape

SKOV3 cells (5×10^3^) were seeded in 35 mm confocal dishes with a glass bottom (Biosharp, China) and cultured overnight. The cells were then incubated with 83.3 μM HA-6(FAM-AS) and ST-6(FAM-AS) for 3 h, incubated with Lyso Tracker Red (Beyotime, China), fixed with 4% paraformaldehyde, and stained with DAPI (Biosharp, China). After incubation with each reagent, the cells were washed 3 times with PBS. Fluorescence imaging was performed using a Nikon ECLIPSE TI (Tokyo, Japan).

### 2.11 *In vitro* imaging in nude mice

Six eight-week-old female nude mice were injected subcutaneously with SKOV3 cells (1×10^6^), and maintained under SPF conditions until the subcutaneous tumor reached 10 mm. The mice were randomly divided into two groups, with three mice per group. HA-6(Cy5-AS) and HA-6(Cy5-AS-like) were injected into mice at a single dose (0.83 μM via the tail vein). The mice were anaesthetized at 1, 2, and 4 h after injection and imaged using a PerkinElmer IVIS Lumina S3 Imaging System (Shanghai, China). *In vitro* imaging of ST-6(Cy5-AS) and ST-6(Cy5-AS-like) was performed after complete metabolism of the HA group.

### 2.12 Statistical analysis

Statistical analyses were performed using the GraphPad Prism (8.0.2, for Windows, GraphPad Software, San Diego, California United States, www.graphpad.com). The data are presented as the mean ± SDs. Welch’s *t*-test was used to compare two groups when the data were normally distributed with uniform variance, and the following *p* values were used to determine statistical significance: n. s., *p* > 0.05; *, *p* < 0.05; **, *p* < 0.01; and ***, *p* < 0.001; ****, *p* < 0.0001.

## 3 Results

### 3.1 Design and characterization of structured aptamers

**FIGURE 1:**
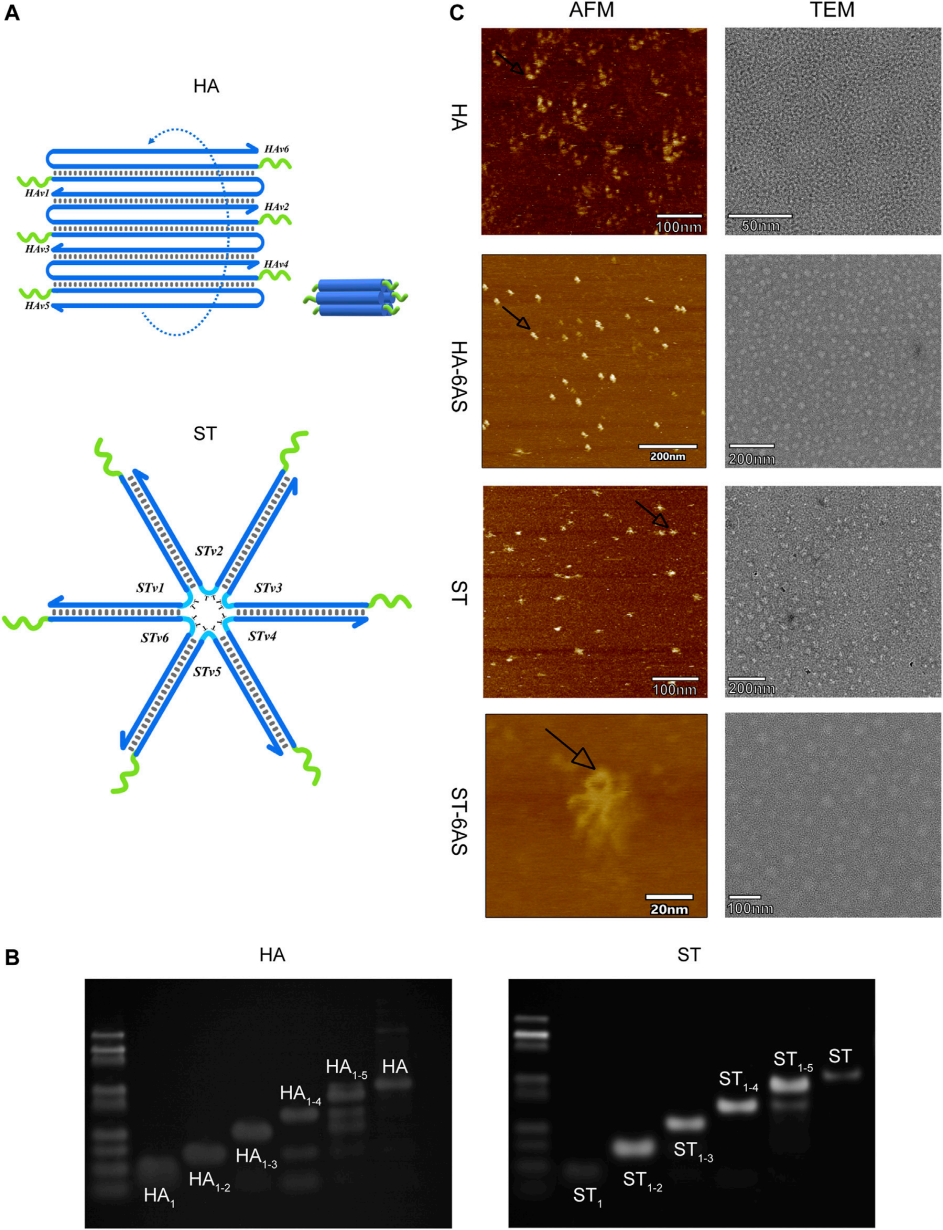
**(A)** Schematic diagram of the structure of HA and ST DNA tiles. The green curve represents the 4 T-deoxcnucleotide spacer and sequences anchored to the AS1411 aptamer handle. The blue curve represents each branch strand of HA or ST. **(B)** PAGE results show the integration of one to six strands of HA and ST branch strands. The most dominant band in the lane is the product. **(C)** AFM and TEM characterization of HA, ST, HA-6AS and ST-6AS. All samples had a concentration of 2 nM. The observed size of ST was about 15 nm, the observed size of HA was about 9 nm. The black arrows indicate the photographed dimer AS1411 aptamer.

HA and ST, designed by NUPACK, are structures that are based on DNA tile self-assembly. Both consist of six DNA single strands with sticky ends that can combine with the handle of the AS1411 aptamers, and these DNA single strands contain repeatable sequences. Inside the anchored sequences of the AS1411 aptamer handle, there is a 4 T-deoxynucleotide spacer (Figure 1A). HA-6AS has a tubular structure with good rigidity, and ST-6AS has a six-star structure with good flexibility. At the turn point of each branch strand, two T-deoxynucleotide transitions provide flexibility to each strand. NUPACK confirmed that both the HA-6AS and ST-6AS structures were thermally stable. Polyacrylamide gel electrophoresis (PAGE) results (Figure 1B; Supplementary Figure S1) showed that HA, ST, and structured aptamers HA-6AS and ST-6AS were successfully self-assembled. The corresponding quantity of AS1411 aptamers that increased gradually from one to six was integrated to demonstrate that HA and ST were successfully connected with six AS1411 aptamers.

The characterization of the two DNA tiles and structured aptamers was performed using AFM and TEM (Figure 1C). The theoretical particle size of ST should be 18 nm, and the theoretical cross-sectional diameter and length of HA should be 7–8 nm. The observed size of ST was approximately 15 nm, and the observed size of HA was approximately 9 nm. The AS1411 aptamers were closer to the HA DNA tiles than the ST tiles. The TEM images showed that HA-6AS and ST-6AS were more homogeneously dispersed than HA and ST. Surprisingly, dimeric AS1411 was photographed for the first time, which corroborated the existence of different forms of AS1411 aptamers.

### 3.2 Serum stability of structured aptamers

The ability of structured aptamers to remain stable in the serum is critical for effective drug delivery. The stability in medium containing 10% FBS at 37°C was evaluated. First, the structured aptamers were incubated in serum for 8 h, and no significant degradation was observed (Supplementary Figure S2). When the incubation time was extended to 24 h (Supplementary Figure S3), HA-6AS and ST-6AS were stable within 12 h, but began to degrade after 24 h.

Recently, DNA tetrahedra (TDN) have been widely used as delivery systems because of their rigid configuration, high flexibility, and ease of synthesis (Fan et al., 2020). Hence, our DNA tiles were compared with TDN (Supplementary Figures S2, S3). The TDN and TDN-AS were also treated in the same way, and the band of TDN nearly disappeared at 24 h, and the band of TDN-AS completely disappeared at 12 h. Consequently, the structured aptamers exhibited improved serum stability.

### 3.3 Determination and quantification of DOX loading

DOX can be loaded into DNA origami structures through intercalation (Zhao et al., 2012; Zhang et al., 2014; Song et al., 2017; Liu et al., 2018; Ge et al., 2020; Pan et al., 2020). After incubation at room temperature, DOX non-covalently conjugated with TDN-AS, HA-6AS, and ST-6AS. A concentration-absorbance standard curve of DOX was established first (Supplementary Figure S4). The loading efficiency and ratio were listed in Table 1. After centrifugation, the DOX concentration in the supernatant was calculated based on absorbance. The drug-loading rate was calculated using Formula 1 (Supplementary Formula S1). The relationship between incubation time and drug loading was then determined (Supplementary Figure S4). No bands were observed in lanes 5 or 8, which were incubated overnight. This could be because more DOX was bound to the structured aptamers, causing the overall positive charge to be excessive, even though the DNA was negatively charged. Structured aptamers loaded with DOX migrated at a slower rate than structured aptamers alone.

**TABLE 1:** Loading efficiency and ratio of three structured aptamers.

Structured aptamers	Loading efficiency (%)	Loading ratio
HA-6AS	69.14	≈1:283
ST-6AS	72.66	≈1:297
TDN-6AS	28.11	≈1:57

### 3.4 Co-localization of DNA carriers and DOX

**FIGURE 2:**
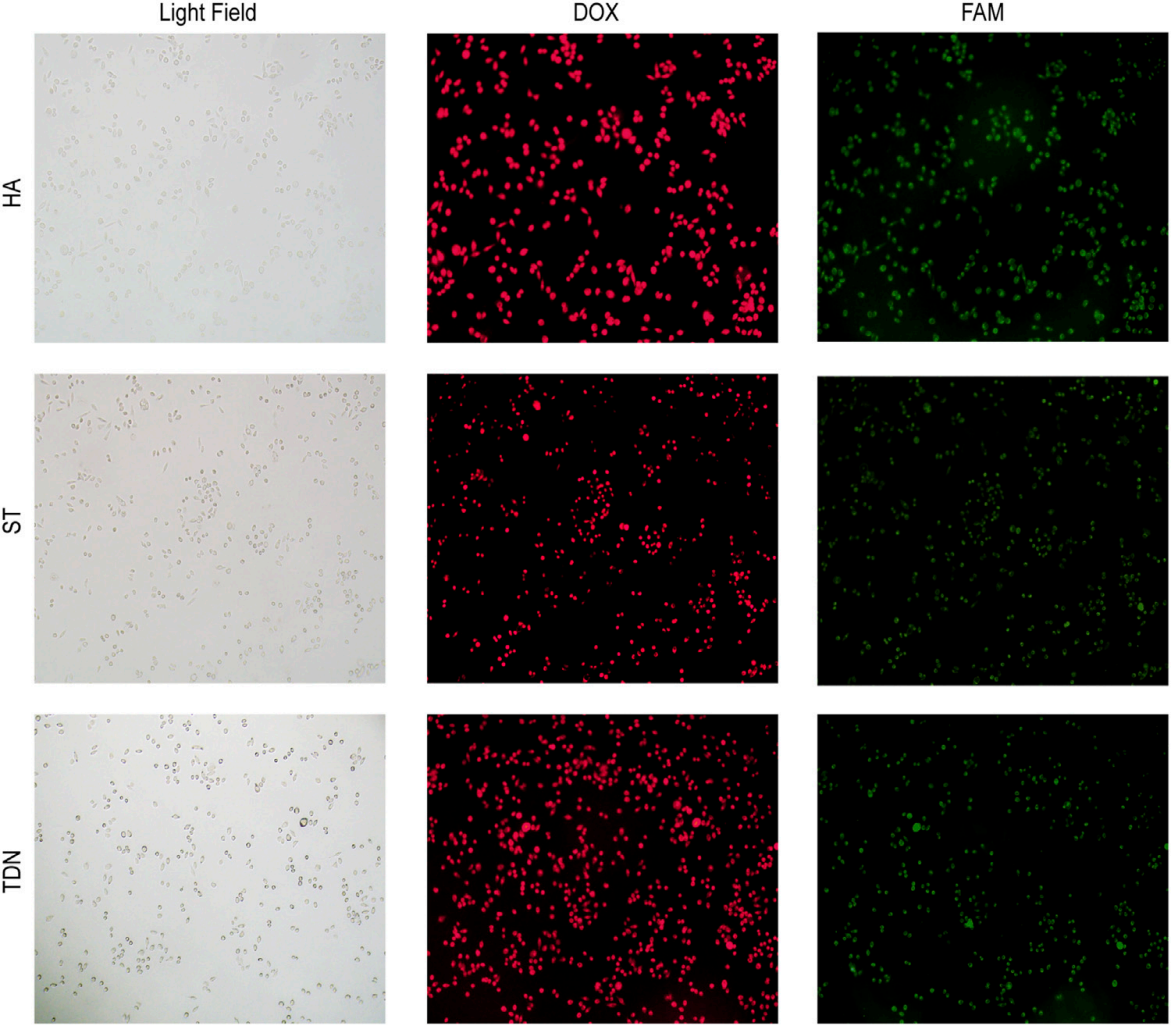
Co-localization results of HA-6AS-DOX, ST-6AS-DOX and TDN-AS-DOX in SKOV3 cells after incubation for 3 h at a concentration of 0.83 μM. The AS1411 aptamers were modified with FAM fluorophore. All images were taken under the same field of view with an inverted fluorescence microscope, the magnification was 100 times. DOX, doxorubicin; TDN, DNA tetrahedra.

To investigate whether HA-6AS and ST-6AS could successfully transport DOX into SKOV3 cells, fluorescence microscopy was performed. After incubating HA-6(FAM-AS) and ST-6(FAM-AS) with DOX overnight, SKOV3 cells were incubated with HA-6(FAM-AS)-DOX or ST-6(FAM-AS)-DOX for 3 h and washed three times before filming with the corresponding field. The fluorescence positions of DOX and FAM were exactly the same as the cellular position (Figure 2), verifying that HA-6AS and ST-6AS could deliver DOX into SKOV3 cells successfully as TDN-AS did.

### 3.5 Uptake of structured aptamers by cells

**FIGURE 3:**
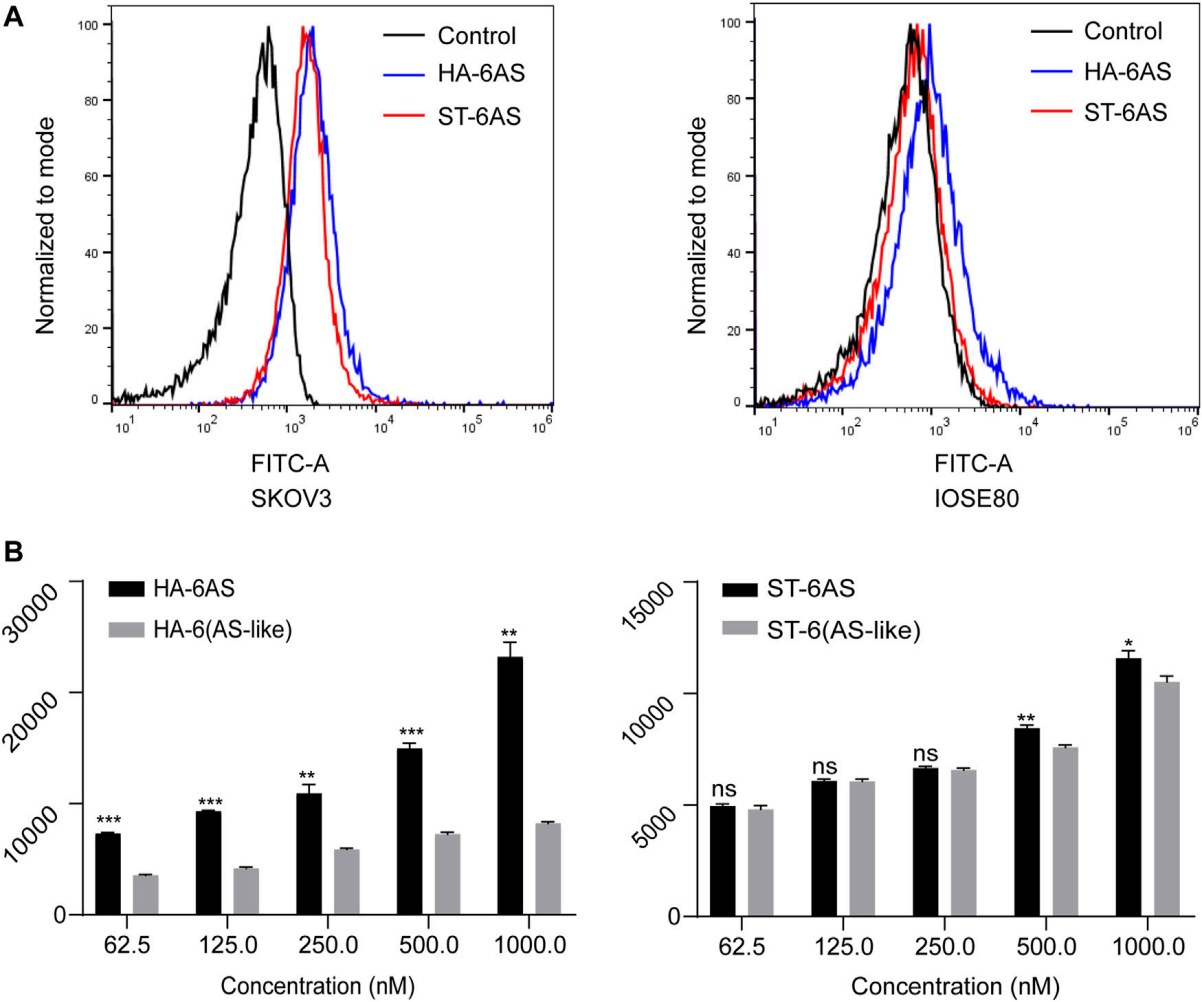
Flow cytometry analysis of the uptake in SKOV3 cells. **(A)** The uptake of HA-6AS and ST-6AS in SKOV3 cells, and the AS1411 aptamers were modified with FAM fluorophore. The SKOV3 cells were incubated with either HA-6AS and ST-6AS at a concentration of 0.83 μM for 3 h. **(B)** The concentration-dependent cellular uptake of the HA-6AS, HA-6(AS-like), ST-6AS, and ST-6(AS-like) in SKOV3 cells at a range of AS1411 concentrations fo r 3 h. The AS1411 aptamers were modified with FAM fluorophore. The data are presented as means ± SDs, *n* = 3, the following *p* values were used to determine statistical significance: ns, *p* > 0.05; *, *p* < 0.05; **, *p* < 0.01 and ***, *p* < 0.001.

To compare the effects of different DNA molecular shapes on the cellular uptake of HA-6AS and ST-6AS, flow cytometry was performed. SKOV3 cells were incubated with HA-6(FAM-AS) and ST-6(FAM-AS) at the same concentration and their uptake was examined by detecting the intensity of FAM fluorescence in the FITC channel. Each sample was measured three times, and 15,000 events were counted. There was no significant difference in the uptake of HA-6(FAM-AS) and ST-6(FAM-AS) by the SKOV3 cells (Figure 3A). In addition, there was no significant uptake of HA-6FAM-AS or ST-6(FAM-AS) by IOSE80 cells. As shown in Figure 3B, the uptake of the two structured aptamers was concentration dependent in SKOV3 cells. To verify the specificity of AS1411 aptamers for cancer cells, a control AS1411 aptamer sequence called AS-like was designed. The length of the AS-like structure was the same as that of AS1411, but it could not form a G-quadruplet. Interestingly, the mean fluorescence intensity of HA-6(FAM-AS-like) was significantly lower than that of HA-6(FAM-AS); however, there were no differences between ST-6(FAM-AS) and ST-6(FAM-AS-like) when the concentration of AS1411 was below 500 nM. Furthermore, the mean fluorescence intensity of ST-6(FAM-AS) was lower than that of HA-6(FAM-AS) at all the concentrations tested.

### 3.6 Cell viability

**FIGURE 4:**
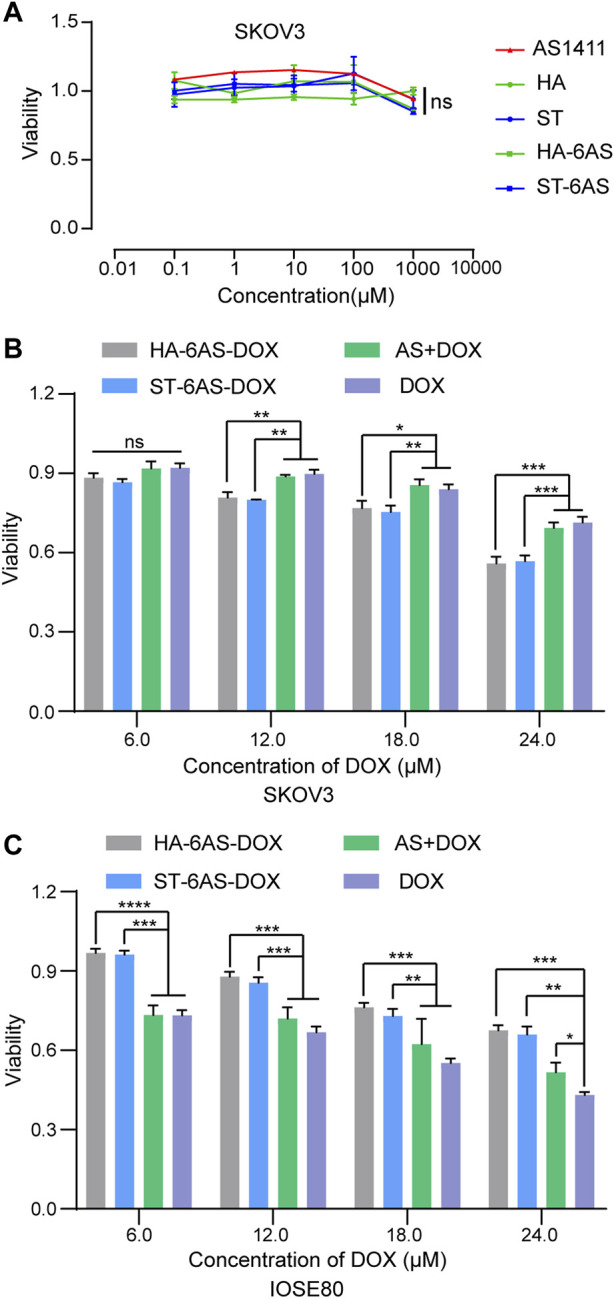
**(A)** Cell viability of SKOV3 cells after 24 h treatment with AS1411, HA, ST, HA-6AS, and ST-6AS evaluated using CCK8 assay. The concentration of each compound ranged from 0.01 to 10,000 μM. The data are presented as the means ± SDs. *n* = 3. **(B)&(C)** Cell viability of SKOV3 **(B)**, IOSE80 **(C)** cells after 3 h treatment with HA-6AS-DOX, ST-6AS-DOX, DOX, AS + DOX evaluated using CCK8 assays. The DOX concentration of each compound ranged from 6 to 24 μM. The data are presented as the means ± SDs. *n* = 3, the following *p* values were used to determine statistical significance: ns, *p* > 0.05; *, *p* < 0.05; **, *p* < 0.01 and ***, *p* < 0.001, ****, *p* < 0.0001.

To examine the cytotoxicity of free AS1411, HA, and ST, HA-6AS, ST-6AS, SKOV3, and IOSE80 cells were incubated with a series of concentrations of these DNA nanostructures for 24 h, and cell viability was detected using the CCK8 reagent (Figure 4A; Supplementary Figure S5). The concentration of free AS1411 ranged from 0 to 1 μM, and the concentration of HA and ST individuals was the same as that of HA-6AS and ST-6AS, of which AS1411 concentration was referred to as free AS1411. There were no obvious losses in the viability of IOSE80 cells when incubated with any of these compounds. To examine the targeting of HA-6AS-DOX, ST-6AS-DOX, SKOV3, and IOSE80 cells were incubated for 3 h with a range of concentrations of HA-6AS-DOX, ST-6AS-DOX, and AS1411 cells with DOX and free DOX. All DOX concentrations were maintained the same in each group (Figure 4B). The viability of SKOV3 cells was visibly lower when incubated with HA-6AS-DOX and ST-6AS-DOX than when incubated with AS1411 cells treated with DOX or free DOX, and there was no statistical difference between HA-6AS-DOX and ST-6AS-DOX. When IOSE80 cells were treated (Figure 4C), the results were completely different. When the incubation time was extended to 24 h, there were no significant differences between DOX-loaded HA-6AS-DOX, ST-6AS-DOX, and free DOX (Supplementary Figure S6). This might be due to the non-covalent binding of DOX and DNA structures; when the incubation time was prolonged, all DOX was fully released into the medium.

### 3.7 Lysosomal escape

**FIGURE 5:**
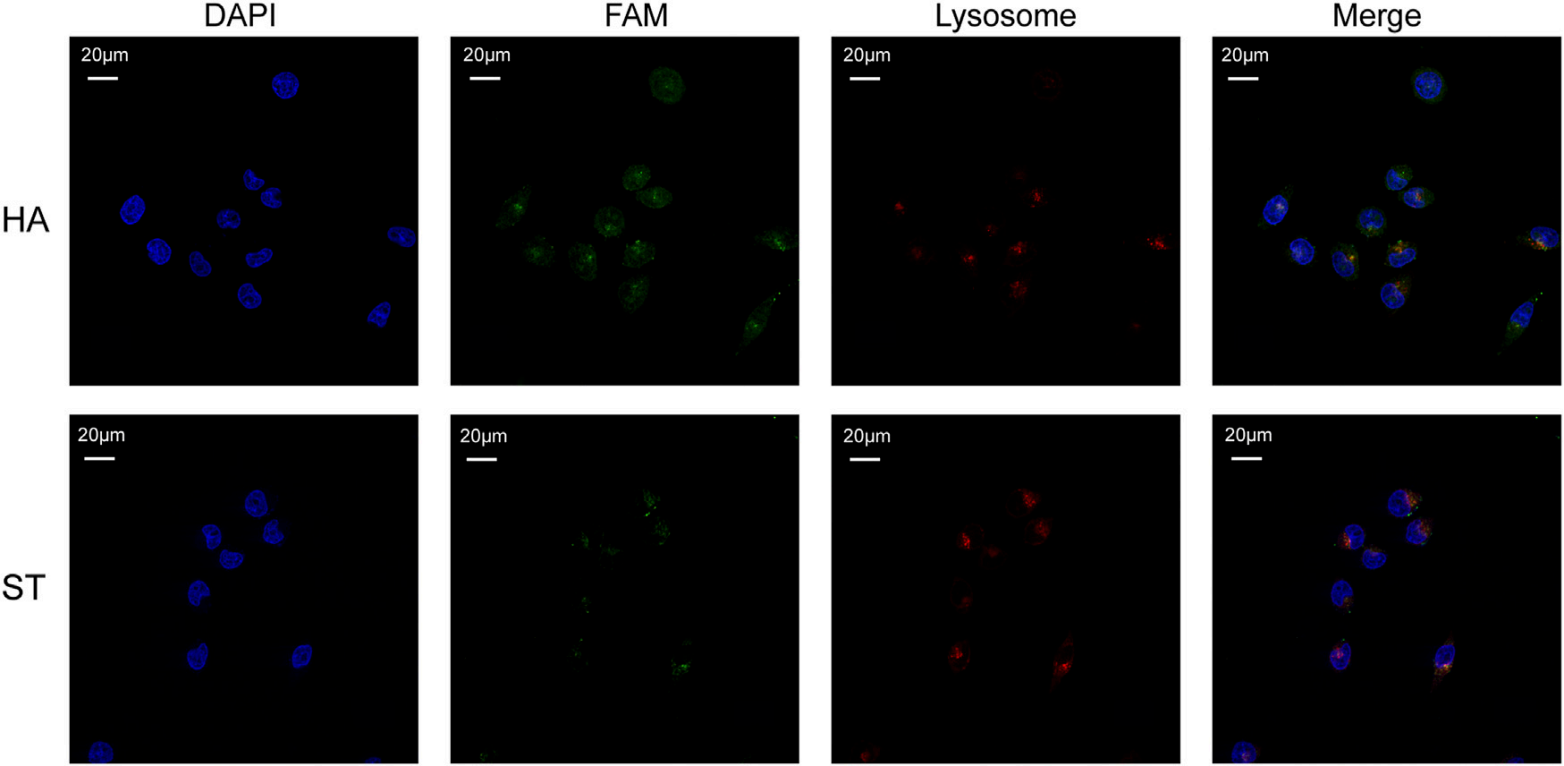
Representative confocal microscopy images showing the co-localization of nuclei (blue), HA-6AS or ST-6AS (green), lysosomes (red) in SKOV3 cells. The nuclei were dyed with DAPI, the AS1411 aptamers were modified with FAM fluorophore, the lysosomes were marked with Lyso Tracker Red. The SKOV3 cells were incubated with HA-6AS or ST-6AS for 3 h, marked with lysosomal tracker, and dyed with DAPI.

Considering the therapeutic mechanism of DOX, it is not desirable for it to be engulfed by lysosomes. At the same time, nucleolin, the target of AS1411, is also expressed in cell nucleus. Therefore, to investigate whether HA-6AS and ST-6AS could deliver DOX into the nucleus and escape lysosomal phagocytosis, confocal laser imaging was performed to analyze the colocalization of HA-6AS or ST-6AS, nuclei, and lysosomes. After incubation with HA-6(FAM-AS) or ST-6(FAM-AS) for 3 h, SKOV3 cells were dyed with Lyso Tracker Red and DAPI, fixed with 4% paraformaldehyde, and imaged. Figure 5 shows the representative confocal images. There was no significant correlation between the position of HA-6(FAM-AS) or ST-6(FAM-AS) and the position of lysosomes. The Pearson’s R values of the fluorescence images were analyzed using Fiji, and the results demonstrated that they weren’t related to colocalization (Supplementary Figure S7).

### 3.8 *In vivo* distribution of the structured aptamers

**FIGURE 6:**
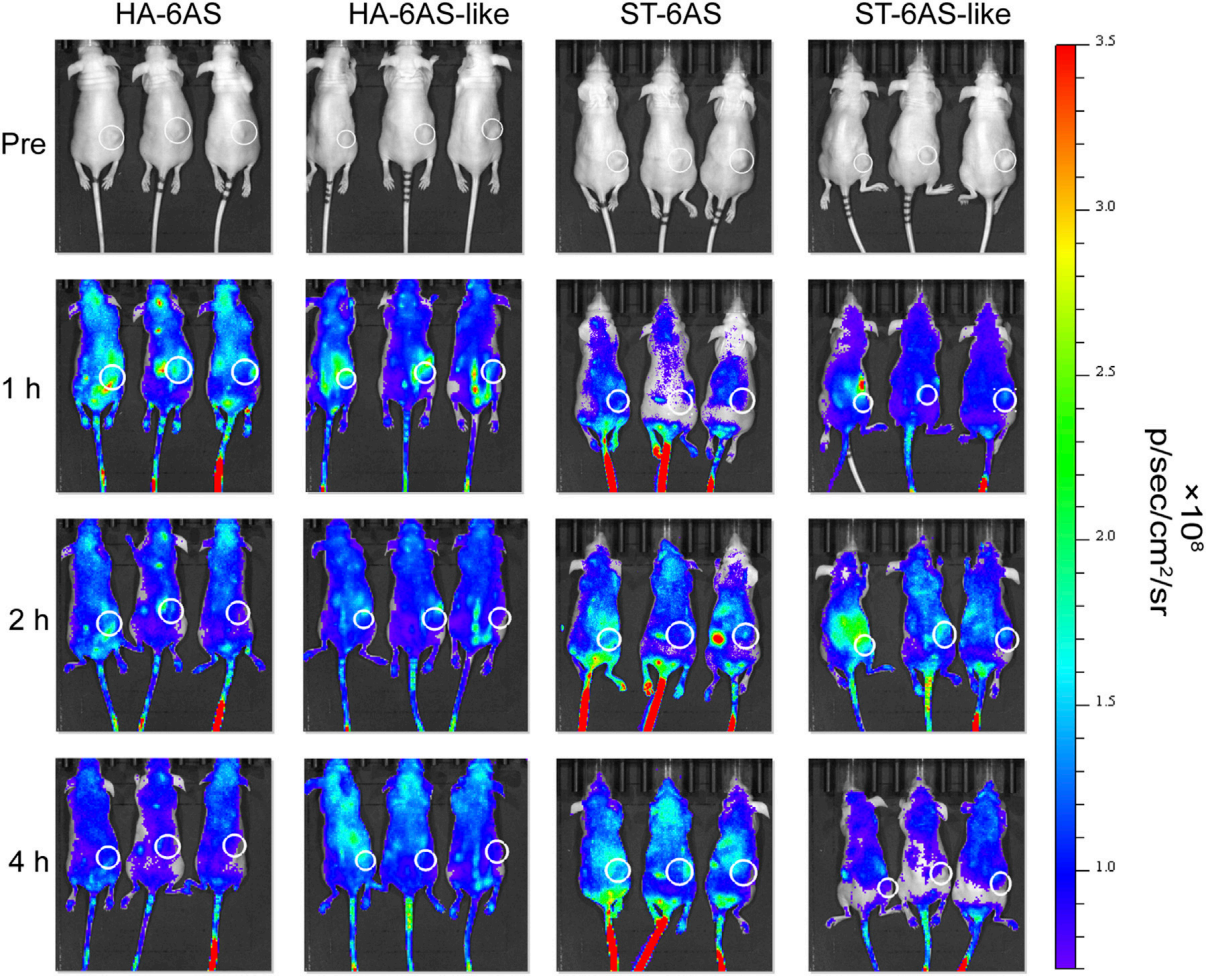
*In vivo* distribution of Cy5 fluorescence in nude mice 1, 2, and 4 h after tail vein injection of HA-6Cy5-AS, HA-6C-AS-like, ST-6Cy5-AS, and ST-6Cy5-AS-like at a concentration of 0.83 μM. The white circles indicate the subcutaneous tumors in nude mice. The scale of fluorescence intensity was consistent for all pictures.

To examine the tumor targeting property of HA-6AS and ST-6AS, *in vitro* fluorescence imaging experiments were performed in nude mice. One, two, and 4 h after tail vein injection, the mice were anaesthetized for whole-body imaging. One hour after injection, the fluorescence intensity of HA-6(Cy5-AS) and HA-6(Cy5-AS-like) in the tumor reached its peak and then gradually declined, and 4 h after injection, the fluorescence completely disappeared in the tumor. The mean fluorescence intensity in the tumor region of HA-6(Cy5-AS) was obviously higher than that of HA-6(Cy5-AS-like) in the first 2 h, then became indistinct. After injection, the fluorescence intensity of ST-6(Cy5-AS) and ST-6(Cy5-AS-like) both reached a peak after 2 h, and ST-6(Cy5-AS) remained until 4 h, but ST-6(Cy5-AS-like) significantly decreased after 4 h. Additionally, the mean fluorescence intensity in the tumor region of ST-6(Cy5-AS) and ST-6(Cy5-AS-like) showed no difference at the beginning, but only became significantly different after 4 h (Figure 6; Supplementary Figure S7).

## 4 Discussion

First, we designed two DNA tiles that could assemble six AS1411 aptamers into specific structures. The experimental results showed that the targeting and drug-loading capabilities of an HA or ST individual significantly outperformed those of TDN. The synthesis of HA-6AS and ST-6AS was simple; only one-pot annealing after mixing was required, and AFM and PAGE results showed that the yield was high. Serum stability experiments also showed that HA-6AS and ST-6AS were more stable for a longer time than TDN-AS. DOX is an anthracycline drug that is beneficial for improving the survival rate in patients with advanced ovarian cancer (*45*) and can be easily loaded into DNA. Therefore, we compared the DOX-loading ability of our DNA tiles and TDN. The experimental results revealed the outstanding loading efficiency and ratio of HA-6AS and ST-6AS. Furthermore, colocalization experiments verified that our structured aptamers could deliver DOX into SKOV3 cells successfully. To explore whether structured AS1411 affects the efficiency of cellular uptake, flow cytometry was performed. SKOV3 cells showed uptake of two types of structured aptamers, but IOSE80 cells didn’t, and cellular uptake of HA and ST was six times more efficient than that of TDN. The uptake of HA-6AS and ST-6AS showed concentration dependence. In addition, the mean fluorescence intensity of ST-6(FAM-AS) was lower than that of HA-6(FAM-AS) at all concentrations tested. The cell viability assays showed that free AS1411 aptamers, HA and ST DNA tiles individuals, HA-6AS, and ST-6AS were non-toxic. Simultaneously, HA-6AS-DOX and ST-6AS-DOX killed SKOV3 cells more rapidly than AS1411 with DOX or free DOX and showed low toxicity to IOSE80 cells. Confocal laser imaging experiments verified that HA-6AS and ST-6AS escaped lysosomal phagocytosis. Finally, *in vitro* fluorescence imaging of nude mice revealed high specificity for HA-6AS, which reached the tumor tissue more quickly than ST-6AS.

The reason for the flow cytometry and *in vivo* fluorescence imaging results may be that the ST structure is prone to passive uptake by ovarian cancer cells. This shows that, even at the same molecular weight, the two structures can differ. The smaller particle size of HA-6AS makes it easier to reach the tumor tissue, whereas ST-6AS takes an unfolded form; therefore, it did not play a good role in the targeting of AS1411 aptamers as the HA structure. Moreover, the mean fluorescence intensity of HA-6AS was higher than that of ST-6AS in these two experiments. From our experimental results, we can easily conclude that the HA structure is more suitable for targeted therapy than the ST structure, which may be because the distance of AS1411 aptamers on the HA DNA tiles is more suitable for the formation of G-quadruplexes. In conclusion, we hope that structured aptamers can further improve the therapeutic effect of chemotherapeutic drugs, such as DOX, and that different aptamers need to be designed with different structures to improve their efficiency.

## Data Availability

The original contributions presented in the study are included in the article/Supplementary Material, further inquiries can be directed to the corresponding author.
