# Supplementary material for: Computable structured aptamer for targeted treatment of ovarian cancer
Source: Front Genet. 2023 May 3;14:1170260. doi: 10.3389/fgene.2023.1170260 (PMC10189780; doi:10.3389/fgene.2023.1170260)
Supplement: Supplementary file 1 [file DataSheet1.pdf]

Supplementary Material

Structured Aptamer for Targeted therapy in Ovarian Cancer

Luoshan Ruan, Liting Han, Xin Chen, *et al.*

*** Correspondence:** xin.li@whu.edu.cn Supplementary Data

Supplementary Figures and Tables

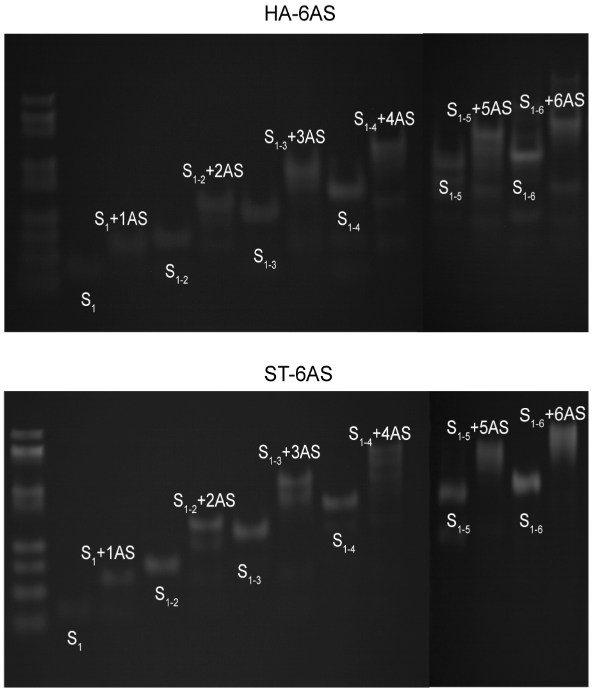

Fig. S1. PAGE results of HA-6AS and ST-6AS. Different amout of branch strands of HA, ST with corresponding AS1411 aptamers.

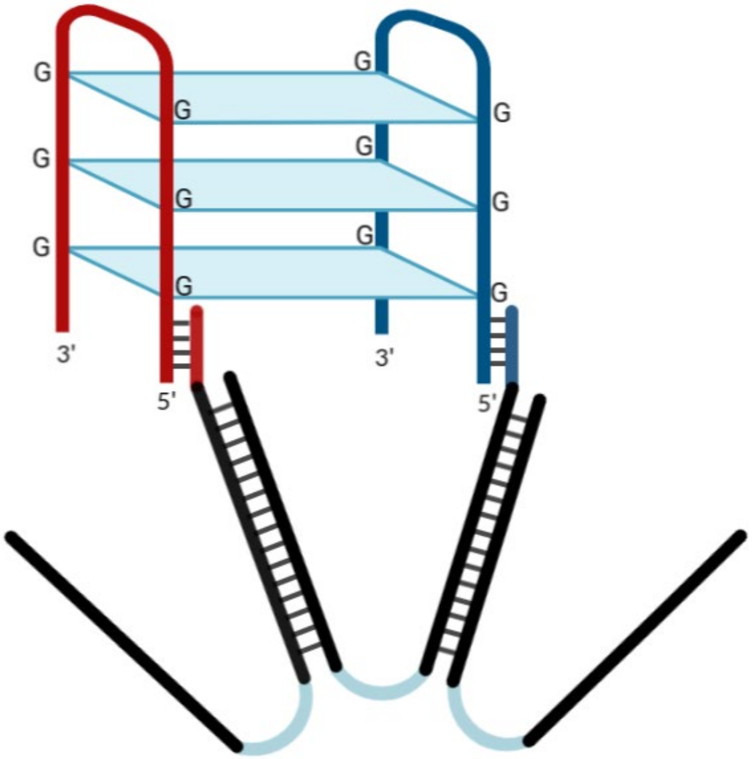

Figure S2. Schematic representation of two AS1411 aptamers forming a parallel dimer structure in ST-6AS as captured by AFM.

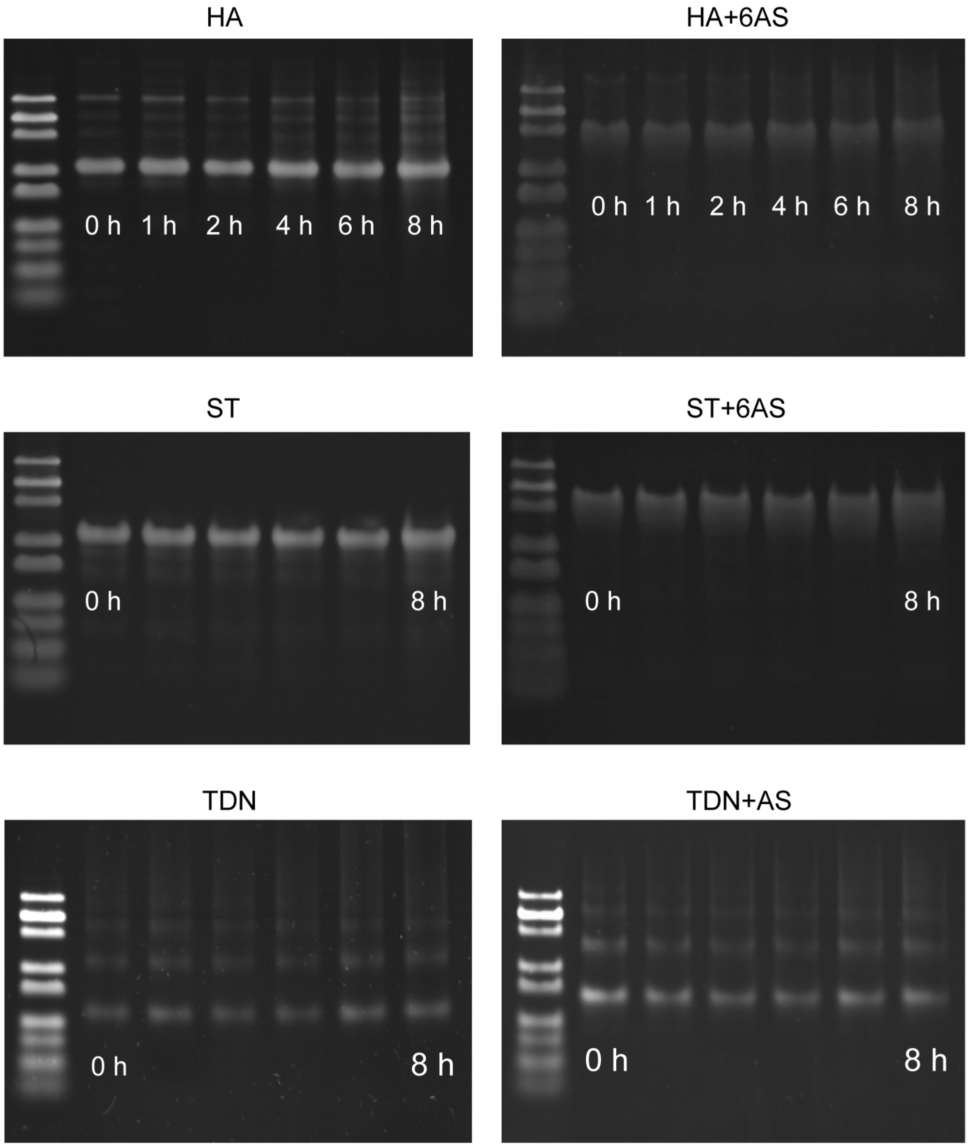

Fig. S3. Serum stability for 8 hours. PAGE results of HA, HA-6AS, ST, ST-6AS, TDN, TDN-AS incubated with 10% serum for 1, 2, 4, 6, 8 hours.

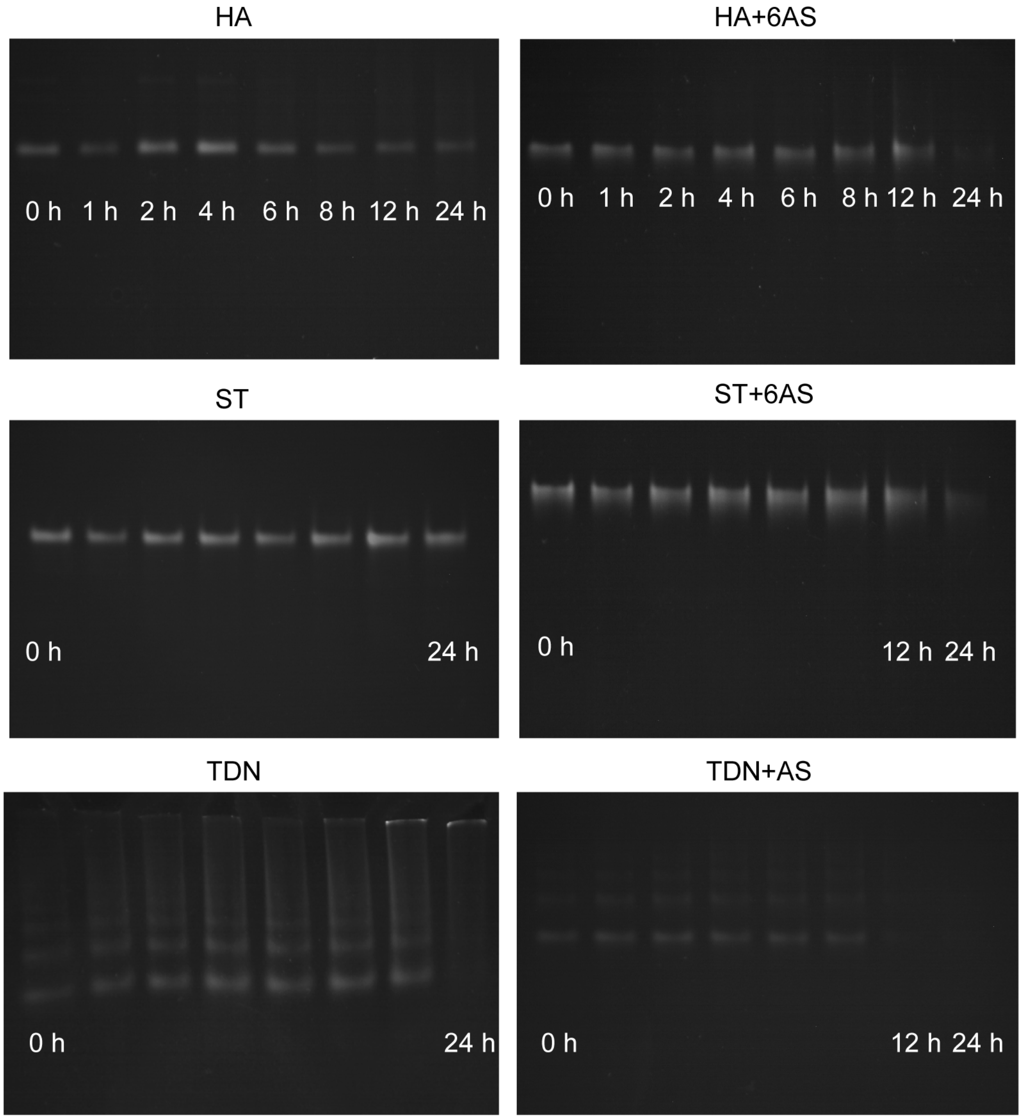

Fig. S4.

Serum stability for 24 hours. PAGE results of HA, HA-6AS, ST, ST-6AS, TDN, TDN-AS incubated with 10% serum for 1, 2, 4, 6, 8, 12, 24 hours.

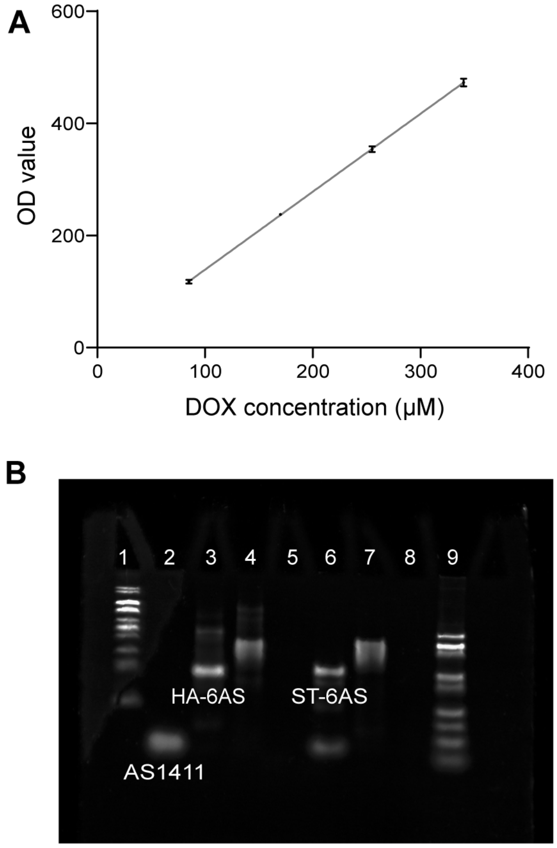

Fig. S5. (A) Concentration-absorbance curve of DOX. The data were present as means ± SDs, n=3. **(B) PAGE results of HA-6AS-DOX and ST-6AS-DOX.** Lane 4 and 7 indicated HA-6AS and ST-6AS incubated with DOX for 1 hour, and lane 5 and 8 indicated HA-6AS and ST-6AS incubated with DOX overnight.

-
- Formula 1:

$$loading\ efficiency = \frac{total\ drugs - free\ durgs}{total\ drugs} \times 100\%$$

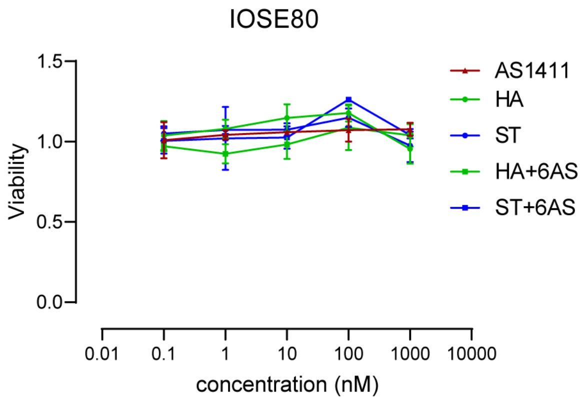

Fig. S6. Cell viability of IOSE80 cells. IOSE80 cells were incubated with a range of concentration of AS1411, HA, ST, HA-6AS and ST-6AS for 24 h. The data were present as means ± SDs, n=3.

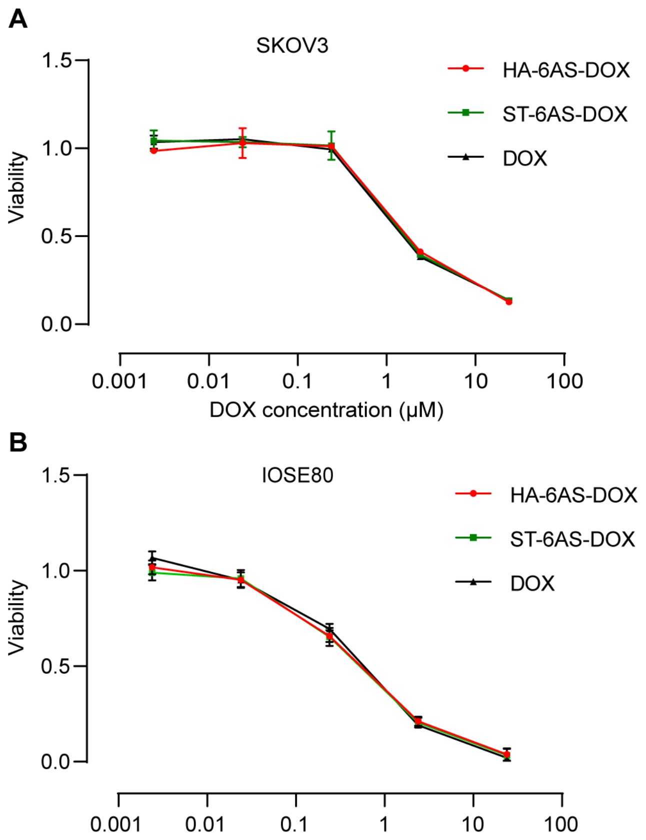

Fig. S7. Cell viability of SKOV3 and IOSE80 cells. SKOV3 and IOSE80 cells were incubated with a range of DOX for 24 h. The data were present as means ± SDs, n=3.

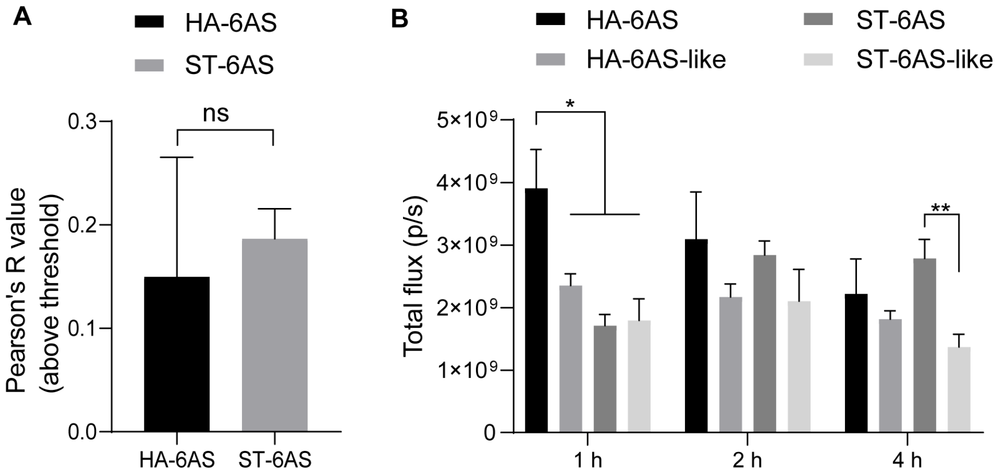

Fig. S8. (A) Pearson's R value (above threshold) of co-localization between the lysosomes and HA-6AS or ST-6AS in SKOV3 cells by Fiji Coloc2. The data were present as means ± SDs, n=3. **(B) Total flux of the circle area in the in vitro fluorescence imaging pictures.** The data were present as means ± SDs, n=3, the following P values were used to determine statistical significance: ns, P > 0.05; *, P < 0.05; **, P < 0.01.

Table S1.

The 5′ to 3′ sequences of branch strands of HA, ST and TDN. The "handle" means the anchored strands to connect with AS1411 aptamer.

Structure	Id	Sequence
HA	HAv1	GTA AGA AAG AAG ATT TTT GGC ATA CAG TCA ACT GTT CGT GCA GGT TTC AGC GAG CGC CG
	HAv2	GTA AGA AAG AAG ATT TTT GCG TCC CCC AAC TCG GAC GCT CGG CGC TCG CTG AAA CCT GC
	HAv3	GTA AGA AAG AAG ATT TTT GCG TCC GAG TTG GGG GAC GCT GCG CTG ACC TCG AGA CGA AC
	HAv4	GTA AGA AAG AAG ATT TTT GCG ACA TTC TAA GTG TCT GCT GTT CGT CTC GAG GTC AGC GC
	HAv5	GTA AGA AAG AAG ATT TTT GCA GAC ACT TAG AAT GTC GCT CGT GCG CCG CGT GGT ACC GC
	HAv6	GTA AGA AAG AAG ATT TTT CGA ACA GTT GAC TGT ATG CCT GCG GTA CCA CGC GGC GCA CG
ST	STv1	GTA AGA AAG AAG ATG AAT TCT CTC TCG CGA CAA GGG TCT GAT CTC ATC GAA AGT GGG CT
	STv2	GTA AGA AAG AAG ATA GCC CAC TTT CGA TGA GAT CAG TTG GAA AAG CTG TGT GAG CAA CT
	STv3	GTA AGA AAG AAG ATA GTT GCT CAC ACA GCT TTT CCA TGC ATA TAT CAA CAT TTA CCG TT
	STv4	GTA AGA AAG AAG ATC ACG GTA AAT GTT GAT ATA TGC TGG CTA CGA CAA AGT CCA CTA TT
	STv5	GTA AGA AAG AAG ATA ATA GTG GAC TTT GTC GTA GCC TGA AGG ATG TAC AGA CAC TAA CT
	STv6	GTA AGA AAG AAG ATA GTT AGT GTC TGT ACA TCC TTC TCC CTT GTC GCG AGA GAG AAT TC
TDN	Handle-S_1	GTA AGA AAG AAG ATA CAT TCC TAA GTC TGA AAC ATT ACA GCT TGC TAC ACG AGA AGA GCC GCC ATA GTA
	S_1	ACA TTC CTA AGT CTG AAA CAT TAC AGC TTG CTA CAC GAG AAG AGC CGC CAT AGT A
	S_2	TAT CAC CAG GCA GTT GAC AGT GTA GCA AGC TGT AAT AGA TGC GAG GGT CCA ATA C
	S_3	TCA ACT GCC TGG TGA TAA AAC GAC ACT ACG TGG GAA TCT ACT ATG GCG GCT CTT C
	S_4	TTC AGA CTT AGG AAT GTG CTT CCC ACG TAG TGT CGT TTG TAT TGG ACC CTC GCA T
